# Improved Cardiac Structure and Ventricular-Arterial Coupling After Baroreflex Activation Therapy

**DOI:** 10.1016/j.jaccas.2025.105351

**Published:** 2025-09-10

**Authors:** Faysal Alhasan, Mark Hoffmann, Melissa Wendell, Renee Kursel, David Lewandowski, German Larrain, Ravi Dhingra

**Affiliations:** a Cardiovascular Division, Department of Medicine, Froedtert and Medical College of Wisconsin, Milwaukee, Wisconsin, USA; b Cardiovascular Division, Aspirus Health, Wausau, Wisconsin, USA

**Keywords:** baroreflex, heart failure, systolic, ventricular remodeling

## Abstract

Baroreflex activation therapy (BAT) improves functional status, quality of life, and exercise capacity in patients with heart failure with reduced ejection fraction; however, its direct effects on reversing adverse cardiac remodeling as assessed by improvements in cardiac structure, function, and coupling with the arterial system remain unclear. We present 2 cases of patients who initially presented with decompensated heart failure, and despite initial medical therapy and continued outpatient follow-up, were unable to tolerate full escalation of guideline-directed medical therapy. The patients remained symptomatic, with high biomarker levels, poor functional capacity, severe heart failure symptoms, and objectively had decreased stroke volume, low left ventricular ejection fraction, and high left ventricular mass. However, soon after implantation of BAT, not only did their heart failure symptoms and hemodynamics improve, but they were able to tolerate further escalation of medical therapy. After BAT, there was evidence of reverse cardiac remodeling, with improvement in biomarkers, left ventricular ejection fraction, contractility, and ventricular-arterial coupling.

## Graphical Abstract

**Figure:**
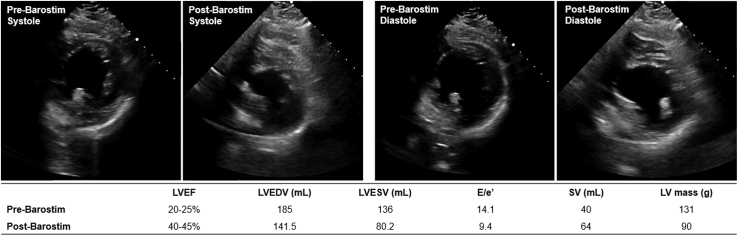

Cardiovascular performance and efficiency are a function of left ventricular contractility and the functional properties of the arterial system. The interaction between the left ventricle (LV) and the arterial system is referred to as ventricular-arterial (VA) coupling, which is quantified as the ratio of the arterial system elastance (E_A_) and LV end-systolic elastance (E_ES_).^1^ In a properly coupled system, left ventricular contractility matches the arterial load, and the VA coupling ratio (calculated as E_A_/E_ES_) is close to 1.00.^1^ In heart failure with reduced ejection fraction (HFrEF), left ventricular contractility is impaired owing to eccentric cardiac remodeling (decreased E_ES_) and an increase in vascular resistance (increased E_A_) resulting in a less compliant arterial system.^2^ The consequential system exhibits VA decoupling (VA coupling ratio >1.00), as the ability of the heart to circulate blood and perfuse peripheral organs is compromised. VA coupling has been demonstrated in several preclinical models, clinical trials, and population-based studies to correlate with progression and prognosis of heart failure, with recent studies showing a strong correlation between noninvasively measured echocardiographic parameters reflective of cardiovascular function and heart failure severity.^3^

**Take-Home Messages**

• The presented cases demonstrate BAT as an effective adjunct therapy for symptomatic treatment of HFrEF in patients who remain symptomatic despite maximally tolerated GDMT.
• These cases demonstrate the potential of BAT to improve cardiovascular performance, as measured by echocardiography, estimated ventricular-arterial coupling, and ability to further uptitrate GDMT.

Reverse cardiac remodeling (RCR) is a dynamic process in which systemic changes to cardiac geometry and function are observed after a period of degradation, resulting in improved cardiovascular performance and patient prognosis. RCR is a therapeutic goal for many heart failure therapies, as it has been shown to improve clinical outcomes. Many efficacious long-term therapies for heart failure have been shown to result in RCR and improved VA coupling.^4^ Baroreflex activation therapy (BAT) has previously demonstrated efficacy in treating symptomatic expression of heart failure in patients with HFrEF, specifically improving functional status, quality of life, and exercise capacity.^5^ Additionally, in experimental studies, BAT improved LV function and partially reversed LV remodeling at both the molecular and cellular levels.^6^ The underlying mechanism of BAT on the autonomic nervous system that restores sympathovagal balance results in systemic physiological changes to promote improvement in HFrEF symptoms such as improved VA coupling, reduced filling pressures, and increased venous capacitance.

The following 2 case studies present evidence of the effectiveness of BAT in improving left ventricular ejection fraction (LVEF), stroke volume, LV mass, and VA coupling.

## Case 1

A 65-year-old Caucasian woman with a history of hypertension, hypothyroidism, and gastroesophageal reflux disease presented to the hospital with acutely decompensated heart failure and NYHA functional class IV symptoms. Her echocardiogram revealed severely reduced LVEF at 10% to 15% as well as mild to moderate functional mitral regurgitation. Initial work-up at an out-of-state facility also included a left heart catheterization that excluded coronary disease; she underwent diuresis, initiated guideline-directed medical therapy (GDMT), and was discharged after 4 days with outpatient follow-up. Ten months later, the patient was readmitted with recurrent decompensated heart failure. Repeat echocardiogram noted a mildly improved LVEF of 20% to 25%. During the hospitalization, GDMT was optimized (see Table 1 for details), and the patient was discharged to a specialized heart failure outpatient clinic after 6 days.

**Table 1:** Case 1: Timeline of Heart Failure Progression, Treatment, and Clinical Variables

Initial diagnosis	Diagnosed with heart failure, with LVEF 10%-15%; family history of cardiomyopathy
10 mo postdiagnosis	Hospitalized for heart failure, with LVEF 20%-25%; on (enalapril) 10 mg daily. NT-proBNP: 558 pg/mL, blood pressure: 115/68 mm Hg, and heart rate: 65 beats/min. Discharged on (carvedilol) 12.5 mg twice daily, (empagliflozin) 10 mg daily, (sacubitril/valsartan) 49/51 mg twice daily, and (spironolactone) 25 mg daily.
11 mo postdiagnosis	Dual-chamber ICD implanted
13 mo postdiagnosis	Started on (losartan) as unable to continue sacubitril/valsartan or empagliflozin owing to cost
14 mo postdiagnosis	Worsening heart failure symptoms, with fatigue and shortness of breath. NT-proBNP was 464 pg/mL. Medical therapy included carvedilol 12.5 mg twice daily, losartan 100 mg daily, and spironolactone 25 mg daily.
15 mo postdiagnosis: Barostim implanted	Implanted with Barostim
1 mo postimplant	Barostim programmed to 4.0 mA
2 mo postimplant	Aldactone held for hyperkalemia. Barostim programmed to 5.8 mA
3 mo postimplant	Barostim programmed to 6.6 mA, and (furosemide) was reduced
4 mo postimplant	Barostim programmed to 7.6 mA with furosemide as needed, and patient reported improved energy. Blood pressure was 128/78 mm Hg, enabling an increase in losartan to 100 mg. NT-proBNP: <36 pg/mL, heart rate: 68 beats/min, and LVEF: 40%-45%.

ICD = implantable cardioverter-defibrillator; LVEF = left ventricular ejection fraction; NT-proBNP = N-terminal pro–B-type natriuretic peptide.

Over the next 3 months, the patient had difficulty tolerating GDMT, particularly sacubitril/valsartan and empagliflozin owing to hypotension, hyperkalemia, and cost. During follow-up, she was noted to be on maximally tolerated GDMT, however she experienced ongoing NYHA functional class III heart failure symptoms, including extreme fatigue. Her clinical status fulfilled the criteria for BAT, with continued heart failure symptoms, LVEF of 20% to 25%, and N-terminal pro–B-type natriuretic peptide (NT-proBNP) of 464 pg/mL.

**Figure 1:**
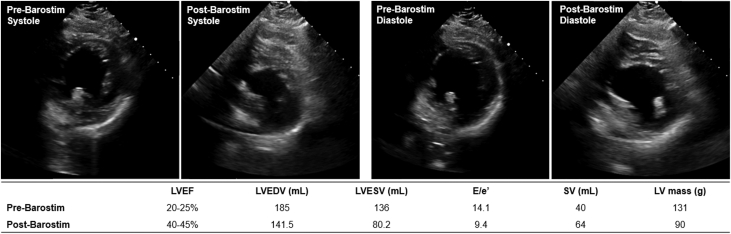
Case 1: Echocardiographic Images of Pre- and Post-Barostim Implant During Systole and Diastole With Associated Clinical Metrics of Interest

One month later, the patient underwent Barostim (CVRx Inc) implantation, with the pulse generator in the right pectoral region and the single lead on the ipsilateral carotid sinus. Chronic BAT began 12 days postimplant, with dose titration occurring monthly through the first 5 months, stabilizing at 7.6 mA. At that point, the patient was noted to have higher blood pressures, with systolic blood pressure running 130 to 140 mm Hg. Her higher blood pressure allowed additional titration of losartan to 100 mg daily, which the patient had been unable to tolerate in the past owing to hypotension. The patient's NT-proBNP was <36 pg/mL, and potassium levels on medical therapy were between 4.0 to 4.5 mEq/L. A repeat echocardiogram showed improvement in ventricular function and structure, with an LVEF of 40% to 45%, reductions in diastolic and systolic volume, an absolute increase in stroke volume of 24 mL, and a 31% decrease in LV mass (Figure 1). Diastolic function as measured by the echocardiographic ratio improved, suggesting normalization of LV filling pressure, and her clinical status improved to NYHA functional class II.

**Figure 2:**
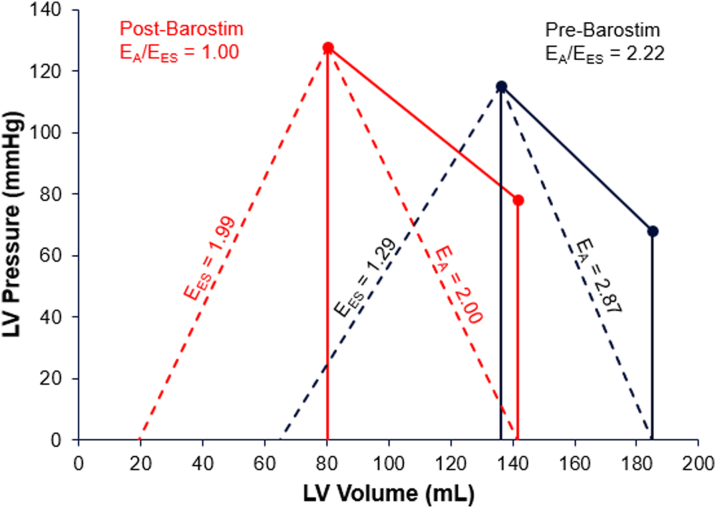
Case 1: Pressure-Volume Relationship Showing Pre- and Post-Barostim Implant Ventricular-Arterial Coupling (E_A_/E_ES_) E_A_ = arterial system elastance; E_ES_ = end-systolic elastance.

Using single-beat end-systolic elastance (E_ES(sb)_) estimation methods,^7^ E_ES_ increased from 1.29 pre-BAT to 1.99 post-BAT, and E_A_ decreased from 2.87 pre-BAT to 2.00 post-BAT. Pre-BAT, the patient had exhibited deteriorating cardiovascular performance as measured by a VA coupling ratio (E_A_/E_ES_) of 2.22, which improved to 1.00 post-BAT (Figure 2).

## Case 2

A 75-year-old man with a history of hypertension, diffuse large B-cell lymphoma treated with 6 cycles of R-CHOP (cyclophosphamide, doxorubicin [>250 mg/m^2^], vincristine, and prednisone), plus the monoclonal antibody rituximab, presented with new-onset heart failure symptoms and atrial fibrillation 1 month after finishing his sixth chemotherapy cycle. His initial echocardiogram revealed a severely reduced LVEF of 20% to 25% compared with a normal LVEF on his prechemotherapy echocardiogram (see Table 2 for details). His new cardiomyopathy was thought to be related to the cardiotoxic effect of anthracycline. He was treated with intravenous diuretics for his decompensated state, and he spontaneously converted to sinus rhythm during hospitalization. He was discharged on only metoprolol succinate 75 mg and bumetanide 1 mg daily, with low systolic blood pressure (<90 mm Hg) as a limiting factor for initiating further GDMT. He was readmitted 3 weeks later owing to acute decompensation of heart failure, underwent further diuresis, and was then discharged on a reduced dose of metoprolol succinate 50 mg, lisinopril 2.5 mg, and bumetanide 1 mg daily.

**Table 2:** Case 2: Timeline of Heart Failure Progression, Treatment, and Clinical Variables

Initial diagnosis	Diagnosed with heart failure, with LVEF 25%-29%, new atrial fibrillation; discharged on bumetanide 1 mg daily and metoprolol succinate 75 mg daily
19 mo postdiagnosis	Hospitalized for heart failure; discharged on bumetanide 1 mg, lisinopril 2.5 mg, and metoprolol succinate 50 mg daily
5 mo postdiagnosis	LVEF: 20%-24%; dual-chamber ICD implanted
6 mo postdiagnosis	Hospitalized for pericarditis and new large pericardial effusion; LVEF 25%-29%
7 mo postdiagnosis	Hospitalized for atrial fibrillation with rapid ventricular response and worsening large circumferential effusion requiring pericardiocentesis; 6MWT distance: 287 m
18 mo postdiagnosis	LVEF: 40%, NT-proBNP: 973 pg/mL, maximally tolerated GDMT consisting of spironolactone 12.5 mg daily, lisinopril 2.5 mg, metoprolol succinate 12.5 mg daily, empagliflozin 5 mg daily, and bumetanide 1 mg as needed
20 mo postdiagnosis: Barostim implanted	Implanted with Barostim, programmed to 4 mA 10 days postimplantation
2 mo postimplant	Barostim programmed to 4.8 mA
6 mo postimplant	Barostim programmed to 5.8 mA; 6MWT distance: 469 m
8 mo postimplant	Barostim programmed to 7.8 mA; decreased need for diuretic use
12 mo postimplant	Barostim programmed to 8.5 mA
19 mo postimplant	Improved appetite and weight; NT-proBNP: 325 pg/mL, LVEF 57%, and 6MWT distance: 492 m

6MWT = 6-minute walk test; GDMT = guideline-directed medical therapy; ICD = implantable cardioverter-defibrillator; LVEF = left ventricular ejection fraction; NT-proBNP = N-terminal pro–B-type natriuretic peptide.

During the next 6 months, he had multiple admissions for pericarditis, atrial fibrillation with rapid ventricular response, gastrointestinal bleed, and cardiac tamponade requiring pericardiocentesis. His LVEF remained severely reduced, with GDMT limited to metoprolol succinate 12.5 mg and lisinopril 2.5 mg owing to bradycardia and hypotension. He was referred to the heart failure clinic, and over the next 6 months his GDMT was titrated to maximally tolerated doses of metoprolol succinate 12.5 mg, lisinopril 2.5 mg, spironolactone 12.5 mg, and empagliflozin 5 mg daily, with bumetanide 1 mg daily. He was euvolemic, with slight improvement of his LVEF, which fluctuated between 25% and 40% dependent on his volume status and rhythm issues but with continued NYHA functional class IIIa heart failure symptoms, fatigue, cardiac cachexia, and elevated NT-proBNP of 973 pg/mL; thus he met indications for BAT.

**Figure 3:**
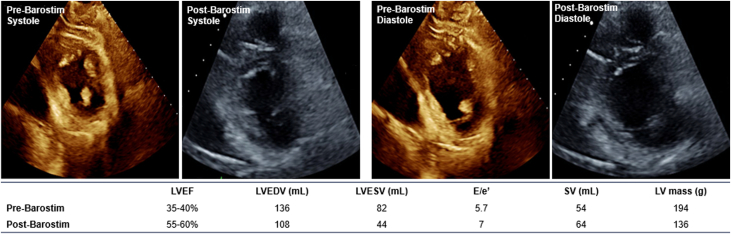
Case 2: Echocardiographic Images of Pre- and Post-Barostim Implant During Systole and Diastole With Associated Clinical Metrics of Interest

Two months later, the patient underwent Barostim implantation, with the pulse generator in the right pectoral region and the single lead on the ipsilateral carotid sinus. BAT started 10 days after implantation and slowly titrated over 1 year to a maximal dose of 8.5 mA. The patient had subjective improvement of his symptoms, with improved appetite, increased weight, and decreased use of diuretics to once every other week. His 6-minute walk test distance also improved to 492.2 m compared with 287 m prior to implantation. His repeat echocardiogram 19 months postimplant showed improvement in ventricular function and structure, with an LVEF to 57%, reductions in diastolic and systolic volume, an absolute increase in stroke volume of 10 mL, and a 30% decrease in LV mass (Figure 3). Along with normalization of both the systolic and diastolic function echocardiographic parameters, NT-proBNP was reduced to 325 pg/mL.

**Figure 4:**
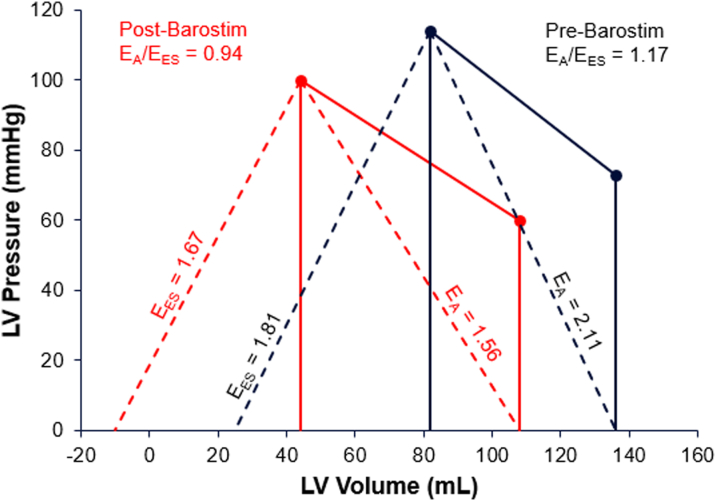
Case 2: Pressure-Volume Relationship Showing Pre- and Post-Barostim Implant Ventricular-Arterial Coupling (E_A_/E_ES_) E_A_ = arterial system elastance; E_ES_ = end-systolic elastance.

Using E_ES(sb)_ estimation methods, E_ES_ decreased from 1.81 pre-BAT to 1.67 post-BAT, and E_A_ decreased from 2.11 pre-BAT to 1.56 post-BAT. The VA coupling ratio (E_A_/E_ES_) was 1.17 pre-BAT and 0.94 post-BAT (Figure 4).

## Discussion

Patients with HFrEF continue to experience poor prognosis and impaired quality of life despite recent advances. The CHAMP-HF registry demonstrated that only 1% of HFrEF patients receive target doses of GDMT, with lower blood pressure being one of the predictors of lower medication use or dosage.^8^ Recent trials such as DAPA-HF and EMPEROR-Reduced have shown that residual risk remains despite high GDMT use, with an annualized event rate of heart failure hospitalization or cardiovascular death of >10 per 100 person-years.^9^ Additionally, therapies such as vericiguat, an oral soluble guanylyl cyclase stimulator, have a role in patients with a systolic blood pressure of >100 mm Hg.^10^ The underuse and intolerance to target doses of GDMT, and the remaining residual risk in optimally treated patients, highlight the unmet need in HFrEF and the role of device-based therapies that can work synergistically with medications, potentially facilitating GDMT tolerance.^11^ Baroreflex activation therapy represents a novel device-based therapy for symptomatic improvement in HFrEF patients with NYHA functional class III or II (who have a recent history of class III) symptoms and a NT-proBNP of <1,600 pg/mL despite being treated with maximally tolerated GDMT. The cases presented describe 2 patients with HFrEF who experienced difficulty tolerating GDMT, with fragile hemodynamic status contributing to the intolerance, and who received BAT.

In case 1, BAT titration over the months after Barostim implantation led to marked improvements in heart failure biomarkers, functional capacity, and NYHA functional classification from III to II within 6 months postimplant. BAT also resulted in a significant improvement in ventricular function and structure as measured by echocardiography (presented in Figure 1). Significant changes in ventricular shape and size were observed, with reductions in diastolic and systolic volumes as well as a 31% decrease in LV mass. These structural changes indicate positive progression along the LV remodeling continuum in response to the improved VA coupling effected by BAT. A notable 15% to 20% improvement in LVEF was also recorded, with an absolute increase in stroke volume of 24 mL. Diastolic function, as measured by the echocardiographic ratio, also improved with BAT, showing a normalization of LV filling pressure. Prior to BAT, the patient exhibited deteriorating cardiovascular performance as measured by a VA coupling ratio of 2.22, indicating progression of heart failure, and unfavorable energetics owing to an increased contribution of potential energy to the pressure-volume area. Improvement in the VA coupling ratio to 1.00 indicates a systemic improvement in cardiovascular performance as observed by changes in cardiac anatomy (described in Figure 1) and provides evidence of the role of BAT in producing systemic changes to improve HFrEF prognosis. Importantly, the increased blood pressure and overall improved hemodynamic status after receiving BAT enabled the patient to tolerate higher doses of GDMT.

In case 2, the patient had anthracycline-induced cardiomyopathy, with initial LVEF of 20% to 24%, and he had multiple early hospitalizations for decompensated heart failure. Despite fluctuating LVEF and limited tolerance of GDMT, he continued to experience limiting symptoms of heart failure, leading to Barostim implantation. After BAT titration, the patient's subjective symptoms improved along with decreased need to use maintenance diuretics. His appetite and weight increased, and his 6-minute walk test distance increased by over 200 m. Objectively, the patient's echocardiogram 8 months post-BAT optimization demonstrated marked improvement in LV structural parameters, with reduction in LV mass, end-diastolic, and end-systolic volumes, alongside normalization of both the systolic and diastolic function echocardiographic parameters (presented in Figure 3).

## Conclusions

These 2 cases present findings that indicate BAT as an effective adjunctive therapy to improve cardiovascular performance in patients with HFrEF who may be unable to tolerate therapeutic levels of GDMT. As observed through structural changes of LV anatomy and improved VA coupling, RCR may be triggered in part by BAT or by the increased GDMT tolerance afforded by BAT. Additional studies are needed to further delineate the effect of BAT on VA coupling and potential RCR.

## Funding Support and Author Disclosures

Ms Wendell has been a speaker for CVRx, Abbott, and Boehringer-Ingelheim. Dr Dhingra has been a consultant for AstraZeneca. All other authors have reported that they have no relationships relevant to the contents of this paper to disclose.
